# BAFF inhibition does not significantly impair immunization responses in patients with rheumatoid arthritis

**DOI:** 10.1186/s13075-015-0867-z

**Published:** 2015-11-30

**Authors:** Clifton O. Bingham, Kevin L. Winthrop, Lili Yang, Chin Lee, Wendy J. Komocsar

**Affiliations:** Division of Rheumatology, Johns Hopkins University School of Medicine, 5200 Eastern Avenue, Mason F. Lord Center Tower Room 404, Baltimore, MD 21224 USA; Divisions of Infectious Diseases, Public Health, and Preventive Medicine, Oregon Health & Science University, 3375 SW Terwilliger Blvd, Portland, OR 97239 USA; Eli Lilly and Company, Indianapolis, IN 46285 USA

Vaccination remains an important strategy in the care of autoimmune disease patients. Patients with rheumatoid arthritis (RA) are at increased risk for infection due to disease-induced immune dysregulation; however, vaccine efficacy can be impaired by concomitant immunomodulators [1, 2]. Tabalumab is a human monoclonal antibody that neutralizes both soluble and membrane-bound B-cell activating factor (BAFF) [3] and was previously investigated for the treatment of RA and systemic lupus erythematosus. While tabalumab development was discontinued following insufficient efficacy observed in phase 3 RA/systemic lupus erythematosus studies, other BAFF pathway drugs are approved for (belimumab) or being investigated in (atacicept, briobacept) other autoimmune indications, and BAFF bi-specific molecules are in development. Given the importance of immunizations to decrease infection risk in autoimmune diseases and the potential for BAFF antagonists to affect responses, we wished to share data from a tabalumab vaccine substudy in RA.

Patients with RA on background methotrexate (MTX) therapy received either a 240 mg loading dose followed by 120 mg of tabalumab monthly (120/Q4W), 180 mg loading dose followed by 90 mg of tabalumab every bi-weekly (90/Q2W), or placebo, and were vaccinated with tetanus, diphtheria, acellular pertussis vaccine (TDaP) and 23-valent pneumococcal polysaccharide (PPSV-23) 24 weeks after drug start. A study flow chart shows this in more detail (Additional file 1). Detailed patient demographic information and study methods are included as Additional file 2 (Methods and Supplemental Table 1). The study protocol was approved by the local institutional review boards in accordance with the Declaration of Helsinki and applicable laws, and all patients provided voluntary written informed consent.

## Findings

**Fig. 1:**
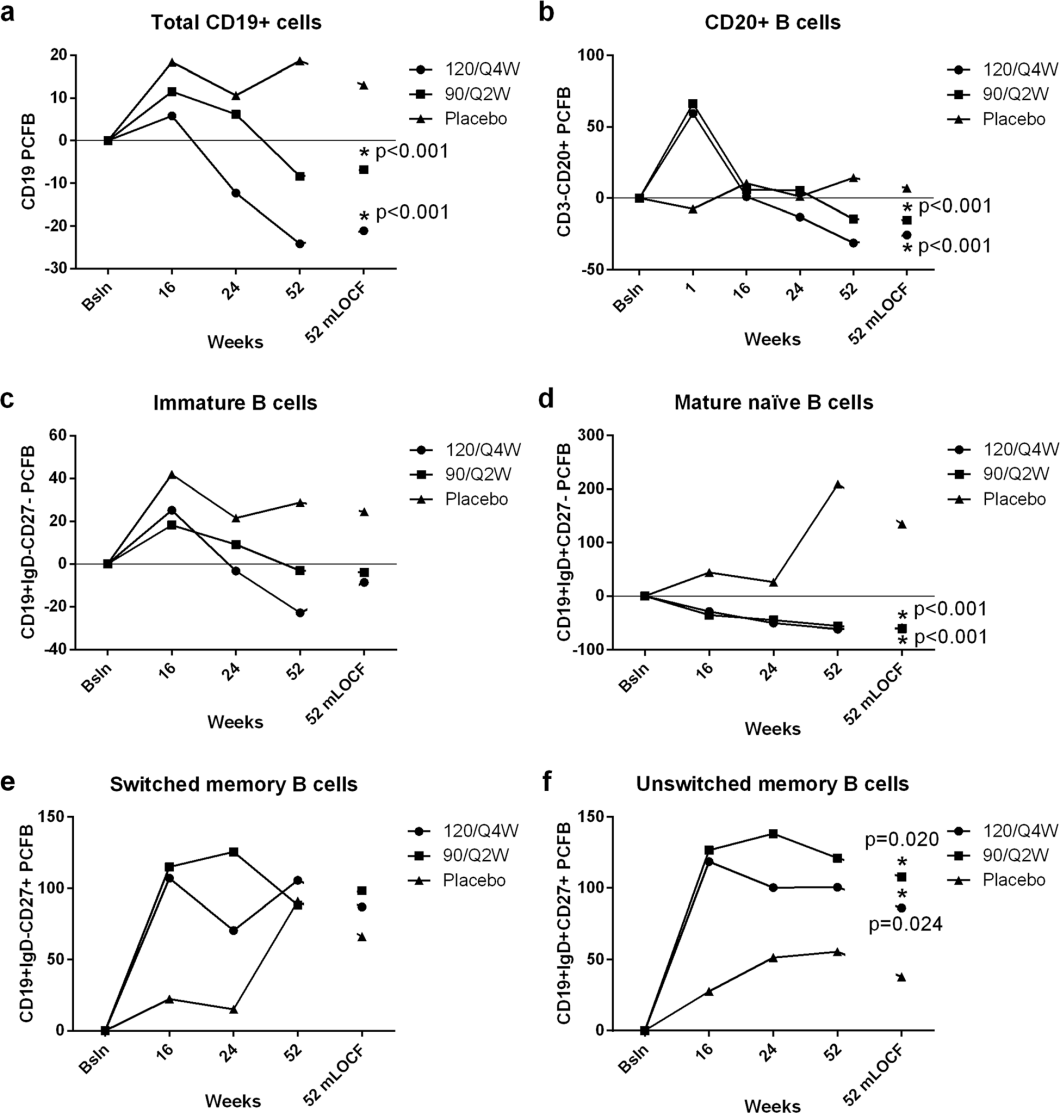
B-cell populations in tabalumab-treated patients versus placebo-treated patients. The percentage change from baseline values in absolute counts of total CD19+ B cells (**a**), CD3-CD20+ B cells (**b**), CD19 + IgD-CD27- immature B cells (**c**), CD19 + IgD + CD27- mature naïve B cells (**d**), CD19 + IgD-CD27+ switched memory B cells (**e**), and CD19 + IgD + CD27+ unswitched memory B cells (**f**) comparing tabalumab-treated (120/Q4W and 90/Q2W) patients and placebo-treated patients. *P* values represent significance in the change from baseline values between tabalumab-treated groups and placebo at week 52 mLOCF (modified last observation carried forward)

Sixty-nine patients completed the vaccine substudy; the substudy was part of a larger 52-week study [4]. Expected reductions in total and naïve B cells and increases in memory B cells were observed (Fig. 1). Total immunoglobulins (Ig) were significantly reduced compared with placebo (Additional file 3). Immunization response data are presented in Table 1. More patients achieved an adequate tetanus IgG response (fourfold or greater increase from baseline) in the 120/Q4W group compared with 90/Q2W or placebo, and the 90/Q2W group was not significantly different from placebo. Further, tabalumab-treated patients had similar responses as placebo in the development of total pneumococcal IgG (twofold or greater increase from baseline). Pre-existing immunity to measles and mumps was also not affected by tabalumab (Supplemental Table 2 in Additional file 2).

**Table 1:** Week 28 (4 weeks post-vaccination) tetanus and pneumococcal antibody immunization responses following 24 weeks of tabalumab treatment

	120/Q4W	90/Q2W	Placebo	*P* value versus placebo	
				120/Q4W	90/Q2W
Tetanus IgG antibody response	*n* = 21^a^	*n* = 30^a^	*n* = 17^a^		
Number of patients with ≥4-fold titer increase where baseline titers ≥0.1 IU/ml^b^ (%)	17 (81.0)	13 (43.3)	10 (58.8)	0.167	0.371
Number of patients with ≥2-fold titer increase where baseline titers ≥0.1 IU/ml^b^ (%)	19 (90.5)	19 (63.3)	10 (58.8)	0.051	0.766
GMT pre-vaccination baseline (95 % CI)	0.301 (0.182, 0.498)	0.491 (0.280, 0.861)	0.341 (0.163, 0.711)		
GMT 4 weeks post-vaccination (95 % CI)	3.495 (1.469, 8.315)	2.216 (1.151, 4.267)	1.963 (0.674, 5.713)	0.263^c^	0.423^c^
Total pneumococcal IgG antibody response	*n* = 21^a^	*n* = 31^a^	*n* = 17^a^		
Number of patients with ≥2-fold titer increase where baseline titers ≥4 mg/L^b^ (%)	15 (71.4)	23 (74.2)	13 (76.5)	>0.999	>0.999
GMT pre-vaccination baseline (95 % CI)	61.78 (46.30, 82.42)	63.77 (50.06, 81.23)	52.53 (36.33, 75.95)		
GMT post-vaccination (95 % CI)	235.62 (150.97, 367.72)	220.81 (147.19, 331.26)	213.04 (121.83, 372.52)	0.835^c^	0.563^c^

*CI* confidence interval, *GMT* geometric mean titer, *Ig* immunoglobulin, *120/Q4W* 120 mg tabalumab every 4 weeks, *90/Q2W* 90 mg tabalumab every 2 weeks

^a^The n values represent the number of patients immunized with vaccine and a baseline and a 28 week antibody titer

^b^Or a titer of ≥0.2 IU/ml for patients with baseline titers <0.1 IU/ml (tetanus) or ≥6 mg/L for patients with baseline titers <4 mg/L (pneumococcus)

^c^ *P* value based on change from baseline log transformed data; rather than providing log transformed titers, we present geometric mean titers as this is the standard way to report these data

Overall this study shows that treatment with tabalumab for 24 weeks did not significantly affect the response to protein or polysaccharide vaccines in RA patients despite expected reductions in B cells and total immunoglobulins.

## Additional files

Additional file 1:
**Supplemental Fig. 1.** Flowchart for study design. Figure showing study design, treatment groups, and timing of immunizations and assessments. (TIF 235 kb) Additional file 2:
**Methods, Supplemental Table 1, Supplemental Table 2, References.** Methods: Description of patient population, study design, endpoints, and analyses. **Supplemental Table 1.** Baseline demographics and disease characteristics of study groups. **Supplemental Table 2.** Geometric mean titers of measles and mumps IgG. References for Methods. (DOCX 30 kb) Additional file 3:
**Supplemental Fig. 2.** Immunoglobulin levels in tabalumab-treated patients versus placebo-treated patients. (TIF 111 kb)

## Abbreviations

BAFF: B-cell activating factor
Ig: Immunoglobulin
MTX: Methotrexate
RA: Rheumatoid arthritis
