# Supplementary material for: BAFF inhibition does not significantly impair immunization responses in patients with rheumatoid arthritis
Source: Arthritis Res Ther. 2015 Nov 30;17:347. doi: 10.1186/s13075-015-0867-z (PMC4665905; doi:10.1186/s13075-015-0867-z)
Supplement: Additional file 2: — Methods, Supplemental Table 1, Supplemental Table 2, References. Methods: Description of patient population, study design, endpoints, and analyses. Supplemental Table 1. Baseline demographics and disease characteristics of study groups. Supplemental Table 2. Geometric mean titers of measles and mumps IgG. References for Methods. (DOCX 30 kb) [file 13075_2015_867_MOESM2_ESM.docx]

# METHODS

Patients. This study examined a subset of 98 patients with RA on background MTX therapy (stable dose 10-25 mg/week), participating in a randomized, double-blind, 52-week study evaluating 2 subcutaneous tabalumab doses (120 mg every 4 weeks [120/Q4W] or 90 mg every 2 weeks [90/Q2W]) vs. placebo (FLEX-M; clinicaltrials.gov: NCT01198002). Patients participating in the study were ≥18 years but <65 years old with moderate to severe RA. This study was conducted in accordance with local institutional review board ethical standards, good clinical practices, and the Declaration of Helsinki. All patients provided written informed consent before study participation.

Patients with moderate to severe active RA were defined as having ≥ 8/68 tender joints and ≥ 8/66 swollen joints, positive for rheumatoid factor (RF) and/or anti-cyclic citrullinated peptide (CCP) antibody at or prior to baseline, had at least one joint erosion due to RA in hand or foot X-rays, and were American College of Rheumatology (ACR) functional class I, II, or III. Only patients who were identified as a treatment responder at week 16, and completed week 24 assessments in the FLEX M study were eligible to participate in the vaccine sub-study. This requirement allowed the assessment of placebo patients that continued on placebo treatment throughout the remainder of the study since week16 placebo non-responders were switched to tabalumab. Patients were excluded from the vaccine sub-study if they met any of the following: history of past vaccination allergy, Arthus-type hypersensitivity reaction following a previous dose of a tetanus-toxoid vaccine within 10 years, anatomical/functional asplenia, or history of Guillain-Barre syndrome. Patients immunized with a tetanus-containing vaccine within the last 5 years or with a pneumococcal vaccine within the last 3 years were excluded due to concerns of an inability to demonstrate an anamnestic response within a short interval between vaccinations [[1](#_http://www.cdc.gov/mmwr/preview/mmw), [2](#_http://www.cdc.gov/mmwr/preview/mmw_1)]. Patients ≥ 65 years old were also excluded from the vaccine sub-study because of the known decrease in immunization response associated with aging [[3](#_Burns_EA,_Lum)]. One patient > 65 years old was inadvertently enrolled; however, the data was not included in the vaccination analyses, only the safety analyses.

During the course of the parent study, the FLEX-M study [[4](#_Smolen_JS,_Weinblatt)] was terminated early due to insufficient efficacy. Therefore, we were unable to enroll the desired number of patients in each treatment group for this vaccine sub-study. Tetanus and pneumococcal immunization responses were examined on the subset of patients that completed the 4-week post-vaccination primary endpoint (28 weeks) and had available baseline titers (n=69). Safety analyses were performed on the total number of patients vaccinated (n=98). Patients were separately consented prior to participating in the vaccination sub-study.

Study Design**.** A subset of patients participating in FLEX-M was immunized at week 24; the study protocol planned to immunize at least 145 subjects; however the study was terminated before completion. Tetanus, diphtheria, acellular pertussis adsorbed vaccine (TDaP) (Boostrix®; GlaxoSmithKline Biologicals, Rixensart, Belgium) and 23-valent pneumococcal polysaccharide (PPSV-23) (Pneumovax 23®; Merck & Company, Inc., Whitehouse Station, NJ) were administered via intramuscular deltoid injection. Anti-tetanus and anti-pneumococcal serum IgG levels were measured at weeks 0 and 24 (pre-immunization), and week 28 (post-immunization) (see Supplemental Figure 1). Pre-existing antibodies to measles and mumps were also measured at weeks 0, 24, and 28. Rubella titers were also collected; however, changes in assay methodology resulted in a small number of patients with titers obtained using the same assay at baseline and later time points. Therefore, given the very low group sizes, the rubella data is not summarized here.

Endpoints. The primary endpoints were the proportion of patients with satisfactory responses 4 weeks after vaccination with TDaP and with PPSV-23. A satisfactory response for the tetanus component of TDaP was defined as ≥ 4-fold increase in anti-tetanus IgG titer in patients with a baseline anti-tetanus IgG titer ≥ 0.1 IU/mL, or ≥ 0.2 IU/mL increase titer in those with a baseline titer <0.1 IU/mL. A satisfactory response to PPSV-23 was defined as ≥ 2-fold increase in anti-pneumococcal capsular polysaccharide IgG titer in patients with a baseline titer ≥ 4.0 mg/L, or ≥ 6.0 mg/L increase in those patients with a baseline titer < 4.0 mg/L [[5](#_Bingham_CO,_III,), [6](#_Bingham_CO,_III,_1)].

Secondary endpoints included the proportion of patients with a ≥ 2-fold increase in anti-tetanus IgG titer compared to baseline in those with a baseline anti-tetanus IgG titer ≥ 0.1IU/mL, or an anti-tetanus IgG titer ≥ 0.2 IU/mL, in those with baseline titers ≤ 0.1 IU/mL. The observed geometric mean titers (GMT) were summarized at each time point for titers of anti-rubeola IgG and anti-mumps IgG.

Descriptions of assays used to measure anti-tetanus, anti-pneumococcal capsular polysaccharide, anti-rubeola and anti-mumps IgG are provided below.

Pharmacodynamics. Samples collected at baseline (week 0), weeks 16, 24, and 52 were evaluated for immunoglobulin levels (IgM, IgG, IgA) and B-cell populations using flow cytometry [total B cells (CD19+ or CD3-CD20+), mature naïve B cells (CD19+IgD+CD27-), switched memory B cells (CD19+IgD-CD27+), unswitched memory B cells (CD19+IgD+CD27+), and immature B cells (CD19+IgD-CD27-)].

Safety. Safety measures in this vaccine sub-study matched those evaluated in the larger FLEX-M study [[4](#_Smolen_JS,_Weinblatt)]. In addition to these parameters, injection site reactions associated with vaccine administration were also recorded.

Statistics. Comparisons between treatment arms were made using Fisher’s exact test for categorical data, analysis of covariance (ANCOVA) with baseline value as covariate, region and treatment arm as fixed effects for B-cell and immunoglobulin (Ig) data, and analysis of variance (ANOVA) for other continuous data. Tests were performed at two-sided significance levels of 0.05. Analyses of GMT change from baseline were performed on log transformed data. Missing data were not imputed for any analyses; for all other patients discontinuing study treatment for any reason, the last non-missing observation before discontinuation was carried forward to subsequent evaluation time points. Vaccine sub-study enrollment was closed early due to parent study termination. The original design planned a sample size of 145 patients (60 patients for each tabalumab group and 25 patients for placebo) to provide ~82% power to detect a reduction of ≥ 32.5% in vaccination response at week 28 between each tabalumab group and placebo, assuming a placebo response rate of 65.0%. Additionally, this sample size would have provided ~87% power to detect a reduction of ≥ 32.5% in vaccination response at week 28 between the pooled tabalumab doses and placebo. The current sample size is about 2/3 of the original sample size.

Assays. The Tetanus Toxoid IgG1 Enzyme Immune Assay (EIA) (The Binding Site, Ltd., Birmingham, UK) was used to measure anti-tetanus IgG. Enzyme-linked immunosorbent assay (ELISA) (Anti-Pneumococcal Capsular Polysaccharide IgG (PCP) ELISA, The Binding Site, Ltd., Birmingham, UK) was used to determine serum levels of anti-pneumococcal capsular polysaccharide IgG in which microwells were pre-coated with 23 anti-pneumococcal capsular polysaccharide antigens (1-5, 6B, 7F, 8, 9N, 10A, 11A, 12F, 14, 15B, 17F, 18C, 19F, 19A, 20, 22F, 23F, 33F-Danish nomenclature). The proportions of patients responding to the aggregate 23 serotypes were comparable across both tabalumab-treated groups and placebo; therefore, individual serotypes were not examined.

The LIASON® Measles IgG assay (DiaSorin Inc., Stillwater, MN), which uses chemiluminescent immunoassay methodology, was used to measure anti-rubeola IgG, and the Mumps IgG ELISA (Gold Standard Diagnostics, Davis, CA) and LIASON® Mumps IgG assay (DiaSorin Inc., Stillwater, MN) were used for the semi-quantitative determination of anti-mumps IgG. During the course of the study, the kit used to measure anti-rubella IgG was changed significantly reducing the data analyzed by the same method for all time points; therefore, anti-rubella data is not included.

# SUPPLEMENTAL RESULTS

## Supplemental Data Table 1: Baseline demographic and disease characteristics of the study groups

| Characteristic | 120/Q4W (n=32) | 90/Q2W (n=41) | Placebo (n=25) |
| --- | --- | --- | --- |
| Age, mean ± SD years (range) | 53.1 ± 10.6 (25-68) | 49.6 ± 9.3 (32-64) | 48.6 ± 9.7 (32-64) |
| Age ≥ 65 years no. (%) | 1 (3.1) | 0 | 0 |
| Female no. (%) | 29 (90.6) | 31 (75.6) | 21 (84) |
| Race no. (%) | | | |
| White | 24 (75) | 24 (58.5) | 14 (56) |
| Black | 2 (6.3) | 1 (2.4) | 1 (4) |
| Asian | 5 (15.6) | 12 (29.3) | 9 (36) |
| Other | 1 (3.1) | 4 (9.8) | 1 (4) |
| Tobacco use no. (%) | | | |
| No | 27 (84.4) | 31 (75.6) | 19 (76) |
| Yes | 5 (15.6) | 10 (24.4) | 6 (24) |
| Time since RA diagnosis (years), mean ± SD years | 6.8 ± 4.7 | 7.1 ± 4.6 | 6.5 ± 4.3 |
| Duration of RA since diagnosis (years), mean (%) | | | |
| 0 to < 2 | 5 (15.6) | 7 (17.1) | 4 (16) |
| ≥ 2 to < 5 | 11 (34.4) | 10 (24.4) | 6 (24) |
| ≥ 5 | 16 (50) | 24 (58.5) | 15 (60) |
| Physician’s assessment of disease activity (VAS) ± SD | 60.8 ± 17 | 61.6 ± 15.5 | 67.5± 18.4 |
| Patient’s assessment of disease activity (VAS) ± SD | 63.1 ± 17.6 | 63.6 ± 20.6 | 65.3 ± 20.2 |
| Patient’s assessment of pain (VAS) ± SD | 62.5 ± 17.9 | 60.5 ± 21.9 | 66.2 ± 20.4 |
| RF positive no. (%) | 0 | 3 (7.3) | 1 (4) |
| Anti-CCP positive no. (%) | 4 (12.5) | 3 (7.3) | 3 (12) |
| Both RF+ and anti-CCP no. (%)+ | 27 (84.4) | 35 (85.4) | 21 (84) |
| Both RF- and anti-CCP- no. (%) | 1 (3.1) | 0 | 0 |
| CRP level, mean ± SD mg/L | 12.1 ± 14.8 | 16.67 ± 21.95 | 16.9 ± 18.94 |
| DAS28-CRP, mean ± SD | 5.55 ± 0.84 | 5.57 ± 0.74 | 5.8 ± 0.93 |
| Tender Joint Count (68 count) ± SD | 23.5 ± 12 | 22.1 ± 11.3 | 25.6 ± 13.9 |
| Swollen Joint Count (66 count) ± SD | 14.5 ± 7.5 | 15.8 ± 10.2 | 17.5 ± 7.9 |
| HAQ-DI score ± SD | 1.54 ± 0.49 | 1.35 ± 0.54 | 1.47 ± 0.8 |
| Disease Severity no. (%) | | | |
| Inactive (DAS28-CRP ≤ 3.2) | 0 | 0 | 0 |
| Moderately active (DAS28-CRP > 3.2 to ≤ 5.1) | 11(34.4) | 11 (27.5) | 7 (28) |
| Very active (DAS28-CRP > 5.1) | 21 (65.6) | 29 (72.5) | 18 (72) |

n = number of patients immunized with tetanus toxoid and pneumococcal polysaccharide vaccines within the treatment groups; RA = rheumatoid arthritis; SD = standard deviation; VAS = visual analogue scale; RF = rheumatoid factor; anti-CCP = anti-cyclic citrullinated peptide; CRP = C-reactive protein; DAS28-CRP = Disease Activity Score (28 joint count and CRP level); HAQ-DI = Health Assessment Questionnaire-Disability Index

## Supplemental Data Table 2: Geometric Mean Titers of Measles and Mumps IgG.

| Anti-rubeola IgG (U/mL) | | | | | P value* vs. Placebo | |
| --- | --- | --- | --- | --- | --- | --- |
|  | 120/Q4W | 90/Q2W | | Placebo | 120/Q4W | 90/Q2W |
| Baseline  (95% CI) | n=23†  276.17  (196.13, 388.89) | n=32  208.45  (139.63, 311.20) | | n=21  146.87  (79.95, 269.81) |  |  |
| Week 24  (95% CI) | n=23  280.60  (203.25, 387.37) | n=32  191.22  (122.77, 297.84) | | n=21  132.90  (67.61, 261.23) | 0.055 | 0.725 |
| Week 28  (95% CI) | n=20  268.88  (185.61, 389.52) | n=32  189.80  (121.52, 296.46) | | n=20  133.97  (66.63, 269.36) | 0.390 | 0.632 |
| Anti-mumps IgG (U/mL) | | | | |  |  |
| Baseline  (95% CI) | n=26  83.74  (48.70, 143.98) | n=32  81.96  (53.32, 125.97) | n=22  146.15  (82.14, 260.04) | |  |  |
| Week 24  (95% CI) | n=24  73.11  (40.34, 132.49) | n=32  85.61  (55.76, 131.42) | n=22  143.61  (81.06, 254.41) | | 0.281 | 0.375 |
| Week 28  (95% CI) | n=20  72.36  (36.57, 143.17) | n=32  77.53  (49.01, 122.65) | n=20  128.10  (67.97, 241.43) | | 0.461 | 0.951 |

*P value based on change from baseline (CFB) log transformed data

†n values represent the total number of patients immunized with TDaP and PPSV-23.

# REFERENCES

## <http://www.cdc.gov/mmwr/preview/mmwrhtml/00047135.htm>. Accessed February 2015.

## <http://www.cdc.gov/mmwr/preview/mmwrhtml/00044572.htm>. Accessed February 2015.

## Burns EA, Lum LG, L'Hommedieu G, Goodwin JS. Specific humoral immunity in the elderly: in vivo and in vitro response to vaccination. J Gerontol 1993;48:B231-B236. doi: 10.1093/geronj/48.6.B231.

## Smolen JS, Weinblatt ME, van der Heijde D, Rigby WF, van Vollenhoven R, Bingham CO, III, et al. Efficacy and Safety of Tabalumab, an Anti-B Cell Activating Factor Monoclonal Antibody, in Patients with Rheumatoid Arthritis who had an Inadequate Response to Methotrexate Therapy: Results from a Phase 3 Multicenter, Randomized, Double-blind Study [abstract]. Ann.Rheum.Dis. 2014;73(Suppl 2):FRI0326.

## Bingham CO, III, Looney RJ, Deodhar A, Halsey N, Greenwald M, Codding C, et al. Immunization responses in rheumatoid arthritis patients treated with rituximab: results from a controlled clinical trial. Arthritis Rheum. 2010;62:64-74. doi:10.1002/art.25034.

## Bingham CO, III, Rizzo W, Kivitz A, Hassanali A, Upmanyu R, Klearman M. Humoral immune response to vaccines in patients with rheumatoid arthritis treated with tocilizumab: results of a randomised controlled trial (VISARA). Ann Rheum Dis. Published Online First: 21 January 2014. doi:10.1136/annrheumdis-2013-204427.
